# Stress hyperglycemia ratio and short-term mortality in critically ill patients with respiratory diseases: a systematic review and dose-response meta-analysis

**DOI:** 10.3389/fmed.2026.1937012

**Published:** 2026-10-08

**Authors:** Wenjie Zhang, Haihong Zhang, Wenxue Zhang, Li Tang

**Affiliations:** 1 School of Nursing, Ningxia Medical University, Yinchuan, China; 2 School of Medical Information and Engineering, Ningxia Medical University, Yinchuan, China

**Keywords:** critical illness, dose-response relationship, meta-analysis, severe respiratory conditions, short-term mortality, stress hyperglycemia ratio

## Abstract

**Objective:**

To systematically evaluate the association between the HbA1c-adjusted stress hyperglycemia ratio (SHR) and short-term mortality in patients with severe respiratory diseases and characterize its linear and nonlinear dose-response relationships.

**Methods:**

PubMed, Embase, China National Knowledge Infrastructure, Wanfang, VIP, and the Chinese Biomedical Literature Database were searched for observational studies evaluating SHR and short-term mortality, including in-hospital, intensive care unit (ICU), 28-day, or 30-day mortality. Odds ratios (ORs) and hazard ratios (HRs) were pooled separately using random-effects models. Sensitivity analyses, linear dose-response analyses, restricted cubic spline analyses, and exploratory analyses of low and high SHR were performed.

**Results:**

Eleven studies were included. Four studies contributed to the OR-based primary meta-analysis. Short-term mortality tended to be higher in the highest SHR category than in the study-specific reference category, but the association was not statistically significant (OR = 2.72, 95% CI: 0.74–9.93, *P* = 0.091; *I*^2^ = 86.4%). Three studies contributed to the HR-based primary meta-analysis, which showed a significant association between higher SHR and increased short-term mortality risk (HR = 1.87, 95% CI: 1.71–2.05, *P* = 0.001; *I*^2^ = 0%). In HR studies, each 0.1-unit increase in SHR was associated with an approximately 5% increase in short-term mortality risk (HR = 1.05, 95% CI: 1.02–1.08, *P* = 0.023). OR studies showed a similar direction of association but did not reach statistical significance. Restricted cubic spline analyses found no clear evidence of nonlinearity. Exploratory analyses suggested higher risks at both low and high SHR levels, but the estimates were imprecise.

**Conclusions:**

Current evidence suggests that higher SHR is associated with increased short-term mortality risk in patients with severe respiratory diseases. HR-based evidence was more consistent and supported a positive linear dose-response relationship. OR-based findings pointed in the same direction but showed substantial heterogeneity and did not reach statistical significance. The available evidence is insufficient to confirm a clear nonlinear pattern. Given the limited number of studies and wide confidence intervals, the apparent increase in risk at both low and high SHR levels should be interpreted as exploratory and does not yet support a stable U-shaped or J-shaped dose-response pattern.

**Systematic Review Registration:**

https://www.crd.york.ac.uk/PROSPERO/view/CRD420261418259, PROSPERO CRD420261418259.

## 1 Introduction

Severe respiratory diseases are a major cause of short-term mortality among hospitalized patients and those admitted to intensive care units (1). In this review, severe respiratory diseases refer mainly to respiratory critical illnesses requiring hospitalization or ICU management, with acute impairment of respiratory function as the main clinical context (2, 3). Common conditions include severe community-acquired pneumonia (SCAP), acute respiratory failure, critical COVID-19, critical chronic obstructive pulmonary disease (COPD) and pulmonary embolism (4, 5). These conditions are often accompanied by hypoxemia, systemic inflammation, circulatory instability and multiorgan injury. Clinical deterioration can be rapid, and mortality risk is high (1, 6, 7). Early identification of high-risk patients is therefore important for clinical decision-making and prognostic assessment. Dysglycemia is a common metabolic feature in acute and critical illness (8). Infection, hypoxia, trauma and inflammation can activate the sympathetic nervous system and the hypothalamic-pituitary-adrenal axis, increase catecholamine and glucocorticoid release, induce insulin resistance and ultimately lead to stress hyperglycemia (9). In the setting of acute hypoxia, infection and systemic inflammation, neuroendocrine stress and insulin resistance may jointly drive acute glucose elevation (10). Previous studies suggest that stress hyperglycemia not only reflects acute disease severity but may also be linked to oxidative stress, endothelial dysfunction, impaired immune function and coagulation abnormalities, thereby contributing to adverse outcomes (11, 12). Glucose-related markers have therefore attracted growing interest in prognostic assessment for critically ill patients.

Admission glucose alone has important limitations (13). It captures an absolute glucose concentration at a single time point and is affected by pre-existing diabetes, chronic glycemic control, dietary intake and therapeutic interventions. As a result, it cannot reliably distinguish acute stress-related glucose elevation from chronic hyperglycemia (14). The same admission glucose level may indicate very different degrees of metabolic stress in a person with long-term normoglycemia and a person with chronically elevated glucose. The HbA1c-adjusted stress hyperglycemia ratio, usually termed the stress hyperglycemia ratio (SHR), is commonly calculated from admission glucose divided by the HbA1c-derived estimated average glucose. It therefore partly adjusts for the individual's chronic glycemic background (13, 15). Compared with admission glucose alone, SHR better reflects the acute deviation of current glucose from the baseline glycemic level and is more suited to capturing relative stress hyperglycemia during acute illness (14). Clinically, an SHR close to 1 indicates that acute-phase glucose is broadly consistent with the long-term average glucose reflected by HbA1c (13, 15). A higher SHR suggests that current glucose exceeds the individual's baseline level and may indicate a stronger acute metabolic stress response.

In recent years, associations between SHR and prognosis in severe respiratory diseases have been reported across populations with SCAP, severe COVID-19, acute respiratory failure, critically ill patients with COPD and pulmonary embolism (16–27). Some studies found that higher SHR was associated with increased risks of in-hospital mortality, ICU mortality or 28-day mortality (19, 21, 22). Others suggested that the relation between SHR and mortality may not be simply linear, but may show U-shaped, J-shaped or threshold patterns, in which both very low and very high SHR are associated with higher risk (19, 23). These findings suggest possible prognostic value for SHR, but individual studies differ in population type, outcome definition, SHR thresholds, reference-group selection and effect-measure reporting. These differences limit direct generalization from single studies.

Previous systematic reviews and meta-analyses of SHR have focused mainly on heart failure (28), acute coronary syndrome (29), stroke or broader cardiovascular disease populations (30). These reviews generally found that higher SHR was associated with increased mortality or adverse prognosis. By contrast, systematic evidence for short-term mortality in patients with severe respiratory diseases remains limited. More importantly, existing studies have often compared the highest SHR group with a reference group, with less attention to low-SHR risk, linear dose-response patterns or potential nonlinearity. Because odds ratios (ORs) and hazard ratios (HRs) have different statistical interpretations, and because reference SHR groups vary across studies, these effect measures should be analyzed separately. We therefore synthesized the evidence from complementary perspectives, including the primary meta-analysis, linear dose-response analysis, and restricted cubic spline (RCS) analysis.

On this basis, we systematically evaluated the association between HbA1c-adjusted SHR and short-term mortality risk in patients with severe respiratory diseases. ORs and HRs were pooled separately to estimate the overall association between higher SHR and short-term mortality. We also performed exploratory analyses of low and high SHR, linear dose-response meta-analyses and RCS analyses to describe the association pattern more fully and to provide evidence for early risk stratification in patients with severe respiratory diseases.

## 2 Methods

### 2.1 Study design and reporting standards

This systematic review and dose-response meta-analysis evaluated the association between the HbA1c-adjusted stress hyperglycemia ratio (stress hyperglycemia ratio, SHR) and short-term mortality risk in critically ill patients with respiratory diseases. The study followed the Preferred Reporting Items for Systematic Reviews and Meta-Analyses (PRISMA) statement (31, 32) and was registered prospectively in PROSPERO (registration number CRD420261418259).

### 2.2 PICO question

The research question was framed using the PICO framework. The population (P) comprised adults with severe respiratory diseases or acute respiratory critical illness. The exposure (I) was SHR, defined as the ratio of acute-phase glucose to the HbA1c-derived estimated average glucose (eAG). The comparator (C) was the study-specific reference SHR category defined in each original study. Because SHR categorization differed across studies, no common numerical threshold was used to define the high-SHR or reference category in the primary meta-analysis. The outcome (O) was short-term mortality, including in-hospital mortality, ICU mortality, 28-day mortality or 30-day mortality. The primary objective was to assess whether higher SHR was associated with increased short-term mortality risk. The secondary objective was to examine whether a dose-response relationship existed between SHR and short-term mortality risk and whether the pattern was closer to linear or nonlinear.

### 2.3 Literature search strategy

We systematically searched PubMed, Embase, China National Knowledge Infrastructure, Wanfang, VIP and the Chinese Biomedical Literature Database from database inception to 1 May 2026. English search terms included “stress hyperglycemia ratio”, “stress hyperglycaemia ratio”, “SHR”, “relative hyperglycemia”, “glycemic gap”, “HbA1c”, “mortality”, “death”, “respiratory failure”, “pneumonia”, “severe community-acquired pneumonia”, “COVID-19”, “chronic obstructive pulmonary disease”, “AECOPD” and “pulmonary embolism”. Chinese search terms included equivalents of stress hyperglycemia ratio, relative hyperglycemia, glycated hemoglobin, severe pneumonia, SCAP, acute respiratory failure, pulmonary embolism, death and prognosis. The complete PubMed search strategy was: (“stress hyperglycemia ratio”[Title/Abstract] OR “stress hyperglycaemia ratio”[Title/Abstract] OR “stress hyperglycemia”[Title/Abstract] OR “stress hyperglycaemia”[Title/Abstract] OR “relative hyperglycemia”[Title/Abstract] OR “relative hyperglycaemia”[Title/Abstract] OR “acute hyperglycemia”[Title/Abstract] OR “acute hyperglycaemia”[Title/Abstract] OR “stress-induced hyperglycemia”[Title/Abstract] OR “stress-induced hyperglycaemia”[Title/Abstract] OR “glycemic gap”[Title/Abstract] OR “glycaemic gap”[Title/Abstract] OR “glucose gap”[Title/Abstract] OR “modified stress hyperglycemia ratio”[Title/Abstract] OR “modified SHR”[Title/Abstract] OR (SHR[Title/Abstract] AND (hyperglycemia[Title/Abstract] OR hyperglycaemia[Title/Abstract] OR HbA1c[Title/Abstract]))) AND (“Pulmonary Disease, Chronic Obstructive”[MeSH Terms] OR “Pneumonia”[MeSH Terms] OR “Respiratory Insufficiency”[MeSH Terms] OR “Pulmonary Embolism”[MeSH Terms] OR “COVID-19”[MeSH Terms] OR “chronic obstructive pulmonary disease”[Title/Abstract] OR COPD[Title/Abstract] OR AECOPD[Title/Abstract] OR “acute exacerbation of chronic obstructive pulmonary disease”[Title/Abstract] OR pneumonia[Title/Abstract] OR “community acquired pneumonia”[Title/Abstract] OR “community-acquired pneumonia”[Title/Abstract] OR “severe community acquired pneumonia”[Title/Abstract] OR SCAP[Title/Abstract] OR “respiratory failure”[Title/Abstract] OR “acute respiratory failure”[Title/Abstract] OR “acute hypoxemic respiratory failure”[Title/Abstract] OR “acute respiratory distress syndrome”[Title/Abstract] OR ARDS[Title/Abstract] OR “pulmonary embolism”[Title/Abstract] OR COVID-19[Title/Abstract] OR SARS-CoV-2[Title/Abstract]) AND (mortality[Title/Abstract] OR death[Title/Abstract] OR prognosis[Title/Abstract] OR survival[Title/Abstract] OR outcome*[Title/Abstract] OR “in-hospital mortality”[Title/Abstract] OR “hospital mortality”[Title/Abstract] OR “ICU mortality”[Title/Abstract] OR “28-day mortality”[Title/Abstract] OR “30-day mortality”[Title/Abstract] OR “short-term mortality”[Title/Abstract]).

### 2.4 Inclusion and exclusion criteria

Studies were eligible for the systematic review if they met the following criteria: (1) adult patients with severe or critical respiratory diseases, including SCAP, critical COVID-19, critically ill patients with COPD or COPD patients admitted to the ICU, acute respiratory failure or pulmonary embolism; (2) the exposure was SHR or another clearly defined HbA1c-adjusted stress hyperglycemia metric calculated from admission glucose and HbA1c-derived average glucose; (3) short-term mortality was reported, including in-hospital mortality, ICU mortality, 28-day mortality or 30-day mortality; (4) the study design was a cohort study, database-based study or dissertation providing sufficient extractable data; and (5) information was available on the association between SHR and mortality.

Studies were included in quantitative meta-analysis only if they additionally met the following criteria: (1) extractable ORs or HRs with 95% confidence intervals (CIs) were reported, or effect estimates could be calculated from the original data; (2) SHR grouping and the reference group were clearly defined; and (3) for dose-response meta-analysis, at least three SHR dose groups were reported with corresponding effect estimates or mortality-event data. Exclusion criteria were: (1) the exposure was not SHR or an HbA1c-adjusted stress hyperglycemia metric; (2) the population was a general ICU or mixed critical-care cohort without separately extractable data for respiratory-disease patients; (3) the outcome was not mortality, or only composite adverse outcomes were reported without extractable mortality data; (4) duplicate publications or studies with overlapping populations or data sources; (5) effect estimates could not be extracted or reasonably calculated; and (6) case reports, reviews, editorials, animal studies, mechanistic experiments and conference abstracts.

### 2.5 Study selection

Two reviewers independently screened the literature. Titles and abstracts were first screened to exclude clearly irrelevant studies. Full texts were then assessed against the inclusion and exclusion criteria to determine eligibility for the systematic review and quantitative meta-analysis. Disagreements were resolved by discussion; persistent disagreements were adjudicated by a third reviewer.

For studies that potentially met inclusion criteria but reported incomplete data, the full text, tables, figures and supplementary materials were checked first. If key data still could not be obtained, the study was included in the narrative synthesis or excluded from quantitative pooling, with the reason recorded.

### 2.6 Data extraction

Two reviewers independently extracted data using a standardized form. Extracted items included first author, publication year, country or region, study design, data source, number of centers, inclusion period, disease type, sample size, age, sex distribution, diabetes prevalence, SHR formula, SHR grouping method, reference-group definition, mortality outcome, follow-up period, effect-measure type, adjusted covariates, OR or HR with 95% CI, numbers of participants and deaths in each dose group, and representative dose values. When both unadjusted and multivariable-adjusted results were reported, multivariable-adjusted estimates were preferentially extracted. When a study reported multiple mortality outcomes, only one effect estimate that best matched the review objective or the original primary outcome was selected for each analysis module, to avoid duplicate inclusion of the same population in the same model. When a study reported multiple SHR comparisons, different comparisons were included in different modules according to the analysis objective, while avoiding duplicate inclusion in the same pooled model. All effect estimates were transformed to the natural logarithmic scale. Standard errors were calculated from the 95% CIs using SE = [ln(upper CI) − ln(lower CI)]/3.92. When the target comparison was not directly reported, transformations were performed on the log scale. If the reference direction in the original article was opposite to the target comparison, the estimate was inverted. If two non-reference groups needed to be compared, the corresponding log effect estimates were subtracted. For Qiao, the low-SHR comparison was obtained by inverting the originally reported OR for Q2 vs. Q1, and the high-SHR comparison was derived from the log OR difference between Q4 vs. Q1 and Q2 vs. Q1. Because this high-end comparison involved a shared reference group and the covariance was not reported, its 95% CI was estimated approximately and was used only in exploratory analysis.

### 2.7 Exposure and outcome definitions

The exposure was SHR, defined as acute-phase glucose divided by the HbA1c-derived eAG, that is, SHR = acute-phase glucose/eAG. When glucose is expressed in mg/dL, eAG (mg/dL) = 28.7 × HbA1c (%) − 46.7. When glucose is expressed in mmol/L, eAG (mmol/L) = 1.59 × HbA1c (%) − 2.59. The latter formula is derived from the former using the conversion 1 mmol/L approximately equals 18 mg/dL. Because the numerator and denominator use the same unit, the two formulae yield equivalent, dimensionless SHR values. We directly extracted SHR groupings, representative doses and effect estimates as reported in the original studies and did not recalculate the original SHR values. For the primary meta-analysis, the highest SHR category reported in each study was compared with the study-specific reference SHR category. Because SHR grouping strategies differed across studies, no common numerical cutoff was applied. For the exploratory analysis comparing both ends of the SHR distribution, an intermediate SHR category was defined as the middle group(s) when a study reported three or more SHR categories, or as the group closest to the median SHR in the study population. This analysis was restricted to studies that provided sufficient data to permit such contrasts. The primary outcome was short-term mortality. In-hospital mortality was prioritized; if it was not reported, ICU mortality, 28-day mortality or 30-day mortality was accepted as short-term mortality. For dose-response analysis, when multiple mortality outcomes were reported, the outcome consistent with the primary analysis was preferentially used.

### 2.8 Methodological quality assessment

Because the included studies were mainly observational cohort studies, methodological quality was assessed using the Newcastle-Ottawa Scale (NOS) (33). The NOS evaluates selection of study groups, comparability of groups and outcome assessment, with a maximum score of 9. Higher scores indicate better methodological quality. Theses were also assessed using the NOS if their design was essentially a retrospective cohort study and if the population, exposure, outcome and extractable effect estimates were clearly defined. The certainty of evidence for the main outcomes was assessed using the GRADE framework. Because this review evaluated prognostic-factor associations rather than intervention effects, the certainty of evidence was assessed considering risk of bias, inconsistency, indirectness, imprecision and publication bias. The detailed GRADE assessment is presented in Supplementary Table S1.

### 2.9 Statistical analysis

In brief, we combined the effect estimates reported by the individual studies into single summary estimates, and we then examined whether the association between SHR and short-term mortality followed a straight-line (linear) or a curved (nonlinear) pattern.

All effect estimates were analyzed on the natural logarithmic scale, and standard errors were back-calculated from the reported 95% CIs. Because ORs and HRs were derived mainly from logistic and Cox regression models, respectively, they have different statistical interpretations. We therefore did not pool ORs and HRs together, but analyzed them separately. Primary meta-analyses used random-effects models, with between-study variance estimated by restricted maximum likelihood. Given the small number of studies in each analysis, confidence intervals were adjusted using the Hartung-Knapp method (34) to obtain more robust interval estimates. Between-study heterogeneity was assessed using *I*^2^ and *τ*^2^, and prediction intervals were calculated when applicable to describe the expected effect range in a future comparable study. Primary meta-analyses were performed separately for OR and HR studies. For categorical analyses, we used the adjusted effect estimate comparing the highest SHR category with the study-specific reference SHR category. The HR primary analysis included independent studies reporting adjusted HRs. When a study reported multiple short-term mortality outcomes, in-hospital mortality was prioritized for the primary analysis; ICU mortality or mortality at other time points was used in sensitivity or supplementary analyses. Studies derived from the same database with potential population overlap were not included together in the same primary analysis. Because Bai (19) and Liao (24) used the same database, the Bai study was retained in the HR primary analysis. Sensitivity analyses assessed the robustness of the primary effects. For OR analyses, pooled estimates were recalculated after excluding the dissertation by Ma (23) and after excluding the COVID-19 study by Mondal (22). For HR analyses, Liao (24) was substituted for Bai (19) to examine the influence of study selection from the same database. Because fewer than 10 studies were included in each quantitative analysis, funnel plots and Egger tests were not used to assess publication bias. Dose-response meta-analyses were also performed separately for OR and HR studies. Linear dose-response analyses pooled study-specific linear trends per 0.1-unit increase in SHR. For studies reporting multiple SHR categories, within-study linear slopes were estimated from dose levels, effect estimates and 95% CIs, and then pooled using random-effects models. When dose-group medians were not reported, closed intervals used the midpoint of the lower and upper limits as the representative dose; open intervals were estimated using adjacent interval widths and distributional information from the original study. Each study was centered at the reference-group dose, the linear slope per unit increase in SHR was estimated on the log-effect scale, and the result was converted to the effect per 0.1-unit increase in SHR. Because some studies did not provide within-study covariance matrices, dose-response results should be interpreted as approximate analyses based on grouped published data. Nonlinear dose-response relationships were assessed using natural cubic spline models, equivalent to RCS modeling in this context. According to the actual analysis settings, spline basis functions were constructed using the ns function from the R splines package with 3 degrees of freedom. This corresponds to a four-knot model with two boundary knots and two internal knots. Boundary knots were set at the minimum and maximum of the dose distribution in each analysis, and internal knots were set at the 33.3rd and 66.7th percentiles. Overall association in the spline model was tested first, and nonlinearity was then assessed by comparing the RCS model with the linear model. The likelihood-ratio test was used as the main test for nonlinearity, with Akaike information criterion used to assess model fit. The OR-based RCS primary analysis included two studies (18, 23), which provided multi-category SHR data suitable for curve assessment. One study reported only three categories (17) and was used in a supplementary analysis. HR-based RCS analysis included three studies (19, 21, 25). To explore whether mortality risk might increase at both ends of the SHR distribution, we performed a separate exploratory analysis in which low- and high-SHR categories were compared with an intermediate SHR category when such contrasts were available or could be derived. This exploratory analysis included only two studies (18, 23). In one study (18), low- and high-SHR estimates were converted from the originally reported grouped ORs, and the results were interpreted as exploratory. All statistical analyses were performed in R, mainly using the meta, metafor and dosresmeta packages. All tests were two-sided, and *P* < 0.05 was considered statistically significant.

## 3 Results

### 3.1 Study selection

The database search identified 540 records. After removal of 5 duplicates, 535 records entered title and abstract screening. After initial screening, 515 records were excluded and 20 reports were assessed in full text. Nine reports were excluded after full-text assessment, mainly because the population did not meet the definition of severe respiratory disease or acute respiratory critical illness, the exposure was not SHR or an HbA1c-adjusted stress hyperglycemia metric, the outcome was not separate short-term mortality, or extractable information on the SHR-mortality association was unavailable for systematic review or quantitative synthesis. Eleven studies were finally included in the systematic review. The study-selection process is shown in Figure 1.

**Figure 1:**
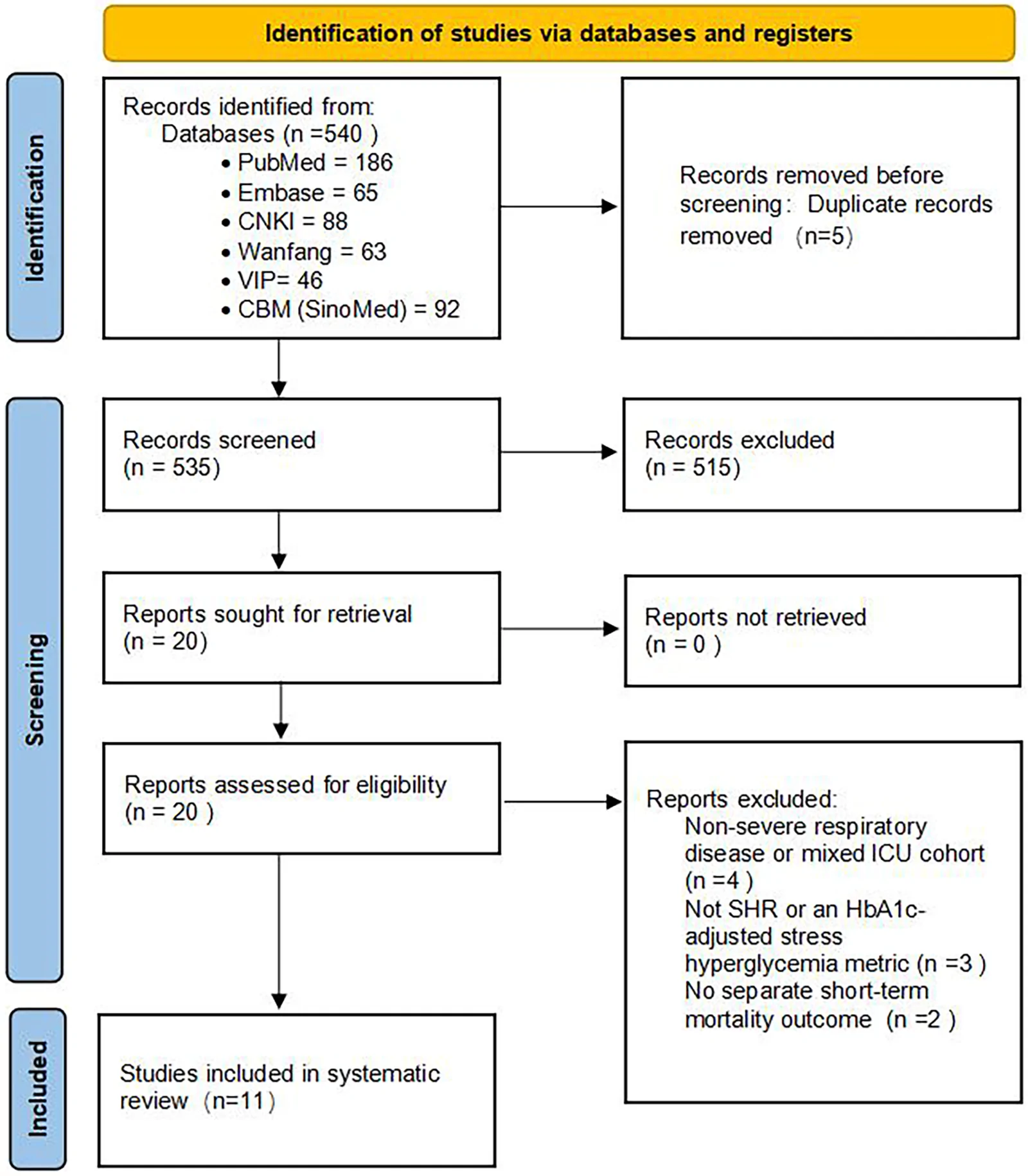
PRISMA 2020 flow diagram for study selection. Adapted from the PRISMA 2020 flow diagram template (32), licensed under CC BY 4.0.

### 3.2 Characteristics of the included studies

All included studies were observational. The populations included patients with severe respiratory diseases or acute respiratory critical illness, such as SCAP, acute respiratory failure, severe COVID-19 and pulmonary embolism. Mortality outcomes included in-hospital mortality, ICU mortality, 28-day mortality and 30-day mortality. SHR definitions, grouping strategies and reference groups varied across studies. Some studies categorized SHR by tertiles or quartiles, whereas others used binary or continuous forms. Study characteristics are summarized in Table 1. According to effect measure and data structure, studies contributed to different quantitative-analysis modules. The OR primary analysis included four studies (17, 18, 22, 23). The HR primary analysis included three studies (19, 21, 25). Because Liao (24) and Bai (19) used the same database and may have overlapping populations, Liao was excluded from the primary analysis and included only in the substitution sensitivity analysis. Studies by Aon (27), Hu (20) and Miao (16) were included in the narrative synthesis but not in the primary meta-analysis because their outcome definitions, exposure grouping or effect-estimate structures were not aligned with the main pooled models. Methodological quality assessment is shown in Table 2.

**Table 1:** Characteristics of the included studies.

First author (year)	Country/data source	Outcome time point	Sample size	Study design	Number of centers	Inclusion period	SHR definition and grouping/modeling	Patient type	Publication type
Aon27	Kuwait; Jahra Hospital	28-day in-hospital mortality	395	Retrospective cohort	Single center	2020-11 to 2021-09	eAG (mmol/L) = 1.59 × HbA1c − 2.59; SHR = admission glucose/eAG; tertiles: ≤0.89, 0.90–1.22, ≥1.23	Hospitalized patients with COVID-19 pneumonia and diabetes	Journal article
Bai19	United States; MIMIC-IV v3.0 (BIDMC)	In-hospital mortality; ICU mortality	2,250	Retrospective cohort	Single-center database	2008–2019	SHR = admission glucose (mg/dL)/(28.7 × HbA1c − 46.7); quartiles, approximately 0.12–0.94, 0.95–1.18, 1.19–1.50, 1.51–15.04	ICU patients with respiratory failure	Journal article
Chen17	United States; MIMIC-IV v2.2 (BIDMC)	In-hospital all-cause mortality; ICU length of stay	1,781	Retrospective cohort	Single-center database	2008–2019	SHR = glucose (mg/dL)/(28.7 × HbA1c − 46.7); tertiles T1/T2/T3, median SHR approximately 0.67, 0.91, 1.33	ICU patients with COPD and type 2 diabetes	Journal article
Du21	United States; NWICU database, 12 Northwestern Medicine hospitals	ICU all-cause mortality; in-hospital all-cause mortality	4,151	Multicenter retrospective cohort	Multicenter	2020–2022	SHR = FBG (mg/dL)/(28.7 × HbA1c − 46.7); SHR quartiles	Critically ill ICU patients with COVID-19	Journal article
Hu20	United States; MIMIC-IV v3.1 (BIDMC)	28-day in-hospital mortality	704 (614 after PSM)	Retrospective cohort	Single-center database	2008–2019	SHR = ABG (mg/dL)/(28.7 × HbA1c − 46.7); RCS-derived cutoff 1.215, categorized as ≤1.215 and >1.215	ICU patients with acute hypoxemic respiratory failure (AHRF)	Journal article
Liao24	United States; MIMIC-IV v2.2 (BIDMC)	28-day ICU all-cause mortality	1,185	Retrospective observational study	Single-center database	2008–2019	SHR = FBG (mg/dL)/(28.7 × HbA1c − 46.7); Q1 ≤ 0.72, Q2 0.72–0.99, Q3: 0.99–1.36, Q4 ≥ 1.36	ICU patients with acute pulmonary embolism	Journal article
Ma23	China; Shengjing Hospital of China Medical University	In-hospital mortality	191	Retrospective study	Single center	2015-11 to 2023-05	eAG (mmol/L) = 1.59 × HbA1c − 2.59; SHR = admission glucose/eAG; G1 < 1.000, G2: 1.000–1.299, G3: 1.300–1.599, G4 ≥ 1.600	Patients with SCAP in a respiratory and critical care unit	Master's dissertation
Miao16	China; Lianyungang Second People's Hospital	28-day mortality	406	Retrospective study	Single center	2020-01 to 2023-12	SHR = admission glucose/eAG; eAG (mg/dL) = 28.7 × HbA1c − 46.7; ROC-derived cutoff 1.14, categorized as <1.14 and ≥1.14	Patients aged ≥65 years with SCAP	Journal article
Mondal22	India; West Bengal HealthWorld Hospitals	In-hospital mortality	451	Prospective observational study	Single center	2021-01 to 2021-12	eAG (mg/dL) = 28.7 × HbA1c − 46.7; SHR = ABG/eAG; SHR ≥ 1.14 defined as elevated	Patients with moderate-to-severe or critical COVID-19, including diabetes, prediabetes and unknown glycemic status	Journal article
Qiao18	China; ICU of a tertiary hospital in Urumqi, Xinjiang	ICU mortality; in-hospital mortality	1,580	Retrospective cohort	Single center	2018-01 to 2023-12	eAG (mmol/L) = 1.59 × HbA1c − 2.59; SHR = ABG/eAG; Q1 ≤ 0.76, Q2: 0.76–0.98, Q3: 0.98–1.50, Q4 > 1.50	ICU patients with COPD	Journal article
Wang25	China; Department of Infectious Diseases, Nanyang Central Hospital	Death from SCAP within 30 days after admission	519	Retrospective study	Single center	2015-01 to 2023-12	SHR = admission glucose/eAG; eAG (mg/dL) = 28.7 × HbA1c − 46.7; T1 < 0.77, T2: 0.77–0.97, T3 > 0.97	Hospitalized patients with SCAP	Journal article

SHR, stress hyperglycemia ratio; eAG, estimated average glucose; ICU, intensive care unit; COPD, chronic obstructive pulmonary disease; SCAP, severe community-acquired pneumonia.

**Table 2:** Methodological quality assessment of the included studies using the Newcastle-Ottawa scale.

Study	Population	Total NOS score
Chen	ICU patients with COPD and T2DM	8/9
Qiao	Critically ill patients with COPD	7/9
Mondal	Moderate-to-severe COVID-19	8/9
Ma	SCAP	7/9
Bai	ICU respiratory failure	8/9
Du	Critically ill COVID-19	8/9
Wang	SCAP	7/9
Liao	ICU pulmonary embolism	8/9
Hu	Acute hypoxemic respiratory failure	7/9
Aon	Hospitalized patients with COVID-19 and diabetes	6/9
Miao	Elderly patients with SCAP	7/9

### 3.3 Primary analysis of the highest SHR category and short-term mortality risk

The OR primary analysis included four studies. Compared with the study-specific reference SHR category, the point estimate for the highest SHR category was above the null, but the confidence interval was wide and crossed 1.0. The random-effects model yielded a pooled OR of 2.72 (95% CI: 0.74–9.93, *P* = 0.091). Between-study heterogeneity was substantial (*I*^2^ = 86.4%, *τ*^2^ = 0.580), and the prediction interval was 0.17–43.02. The OR forest plot is shown in Figure 2A.

**Figure 2:**
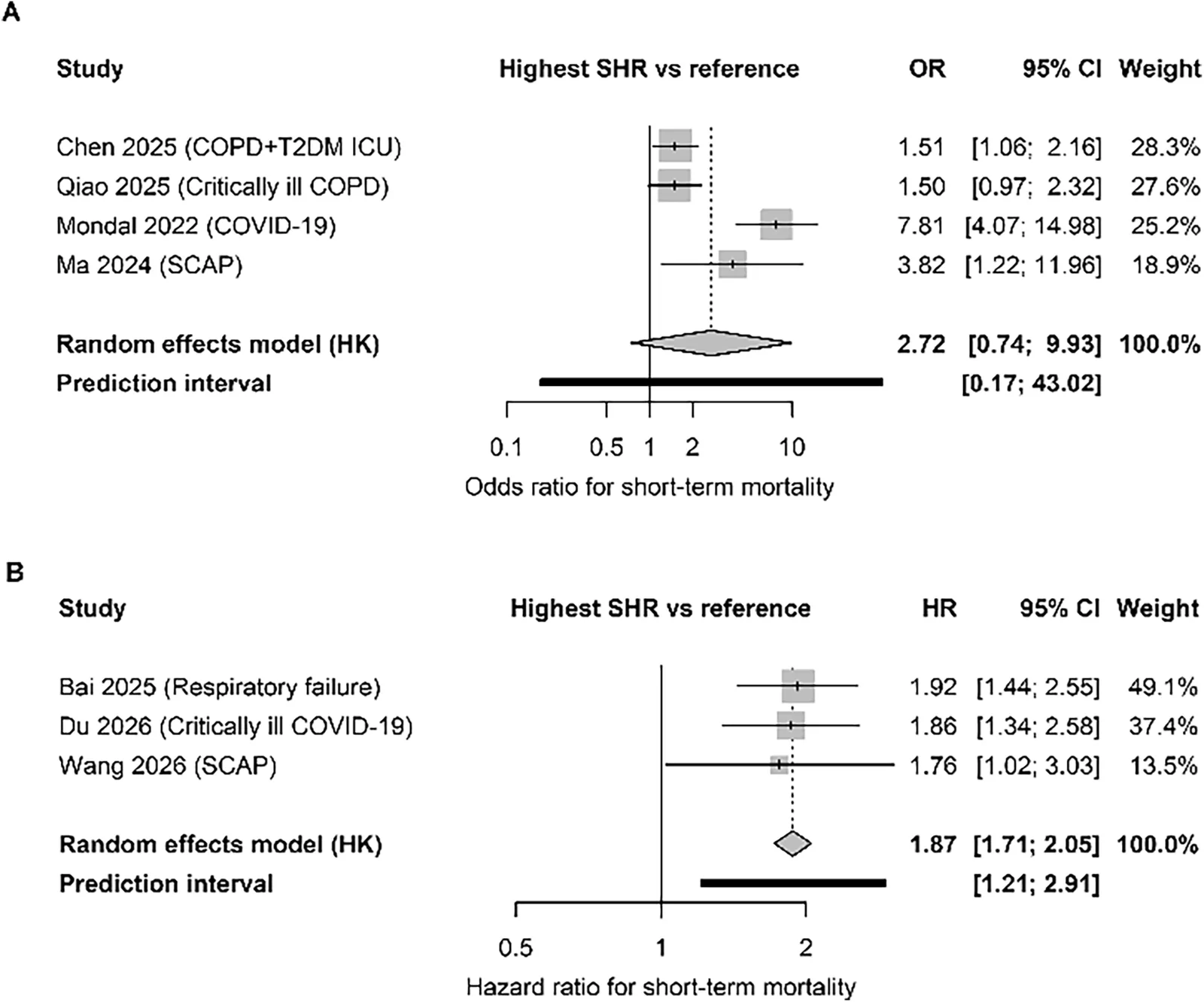
Forest plots of the association between the highest SHR category and short-term mortality relative to the study-specific reference SHR category. **(A)** Odds ratio studies. **(B)** Hazard ratio studies.

The HR primary analysis included three independent studies. Higher SHR was significantly associated with increased short-term mortality risk. The pooled HR was 1.87 (95% CI: 1.71–2.05, *P* = 0.001). No evident between-study heterogeneity was observed (*I*^2^ = 0%, *τ*^2^ = 0), and the prediction interval was 1.21–2.91. The HR forest plot is shown in Figure 2B.

Overall, the OR and HR point estimates were in the same direction, although only the HR-based primary analysis reached statistical significance. OR studies showed substantial heterogeneity and wide confidence intervals, whereas HR studies yielded more consistent estimates, showed no evident statistical heterogeneity and reached statistical significance. Primary-analysis results are summarized in Table 3.

**Table 3:** Summary of the main-effect and sensitivity meta-analyses.

Analysis	Measure	*k*	Pooled estimate	95% CI	*P* value	*I*^2^ (%)	*τ* ^2^	Prediction interval
OR main analysis	OR	4	2.72	0.74–9.93	0.091	86.4	0.580	0.17–43.02
HR main analysis	HR	3	1.87	1.71–2.05	0.001	0.0	0.000	1.21–2.91
OR sensitivity excluding Ma dissertation	OR	3	2.53	0.25–25.98	0.228	90.4	0.792	0.03–220.10
OR sensitivity excluding Mondal COVID-19	OR	3	1.59	0.83–3.03	0.092	17.2	1.5 × 10^−6^	0.88–2.86
HR sensitivity replacing Bai with Liao	HR	3	2.43	0.71–8.32	0.090	83.0	0.203	0.24–24.21

CI, confidence interval; HK, Hartung-Knapp adjustment; *I*^2^, inconsistency; *τ*^2^, between-study variance. Prediction intervals describe the expected range for a future comparable study.

### 3.4 Sensitivity analyses

To assess robustness, sensitivity analyses were performed. In OR analyses, exclusion of the dissertation by Ma (23) yielded a pooled OR of 2.53 (95% CI: 0.25–25.98, *P* = 0.228, *I*^2^ = 90.4%). The point estimate remained above 1.0, but the confidence interval became markedly wider, indicating that Ma (23) was not the sole source of heterogeneity in the OR primary analysis. After excluding the COVID-19 study (22), the pooled OR was 1.59 (95% CI: 0.83–3.03, *P* = 0.092, *I*^2^ = 17.2%). Compared with the OR primary analysis, heterogeneity decreased substantially, suggesting that the COVID-19 study contributed to heterogeneity in the OR pooled estimate and that effect sizes may differ across respiratory-disease subtypes. In HR analyses, replacing Bai (19) with Liao (24) yielded a pooled HR of 2.43 (95% CI: 0.71–8.32, *P* = 0.090, *I*^2^ = 83.0%). The point estimate remained above 1.0, but between-study heterogeneity increased markedly. This sensitivity analysis indicates that the pooled HR was sensitive to the choice of database-derived study. Sensitivity-analysis results are shown in Table 3.

### 3.5 Linear dose-response analysis

The OR linear dose-response analysis included three studies. For each 0.1-unit increase in SHR, the pooled estimate was above 1.0, but the confidence interval crossed the null and the result did not reach statistical significance. The pooled OR was 1.03 (95% CI: 0.97–1.10, *P* = 0.162, *I*^2^ = 0%). These results are compatible with a possible positive linear association in OR studies, but the current evidence is insufficient to support a statistically significant linear association.

The HR linear dose-response analysis included three studies. Each 0.1-unit increase in SHR was significantly associated with increased short-term mortality risk. The pooled HR was 1.05 (95% CI: 1.02–1.08, *P* = 0.023, *I*^2^ = 0%). These findings are compatible with a positive linear dose-response association in HR studies. Linear dose-response results are shown in Table 4.

**Table 4:** Dose-response, restricted cubic spline, and exploratory low/high SHR analyses.

Analysis	Measure	*k*	Summary estimate/test	95% CI	*P* value	*I*^2^ (%)	Nonlinearity P
OR linear dose-response per 0.1 SHR	OR	3	1.03 per 0.1 SHR increase	0.97–1.10	0.162	0.0	–
HR linear dose-response per 0.1 SHR	HR	3	1.05 per 0.1 SHR increase	1.02–1.08	0.023	0.0	–
OR RCS main: Qiao + Ma	OR	2	Overall RCS association	–	0.051	–	0.941
OR RCS supplementary: Chen + Qiao + Ma	OR	3	Overall RCS association	–	0.008	–	0.822
HR RCS main: Bai + Du + Wang	HR	3	Overall RCS association	–	0.013	–	0.736
Low SHR vs. intermediate SHR category exploratory analysis	OR	2	2.20	0.08–57.69	0.200	4.8	–
High SHR vs. intermediate SHR category exploratory analysis	OR	2	3.11	0.64–15.15	0.070	0.0	–

RCS, restricted cubic spline; SHR, stress hyperglycemia ratio. Nonlinearity *P* values compare the RCS and linear models. For Qiao, low/high SHR exploratory comparisons were converted from the originally reported grouped ORs; the CI for the high-SHR comparison was approximated because covariance between shared-reference contrasts was unavailable.

### 3.6 Nonlinear dose-response analysis

The exploratory OR-RCS analysis included two studies (18, 23). The overall association in the spline model did not reach statistical significance (*P* = 0.051), and the RCS model did not fit better than the linear model (*P* for nonlinearity = 0.941). Thus, the current evidence does not support a clear nonlinear relationship between SHR and short-term mortality risk in OR studies. The OR dose-response curve is shown in Figure 3A. In the supplementary analysis including Chen (17), the overall association in the OR spline model reached statistical significance (*P* = 0.008), but the nonlinearity test remained non-significant (*P* = 0.822). This suggests that adding a three-category study strengthened the overall association but did not change the conclusion that no clear nonlinear pattern was observed.

**Figure 3:**
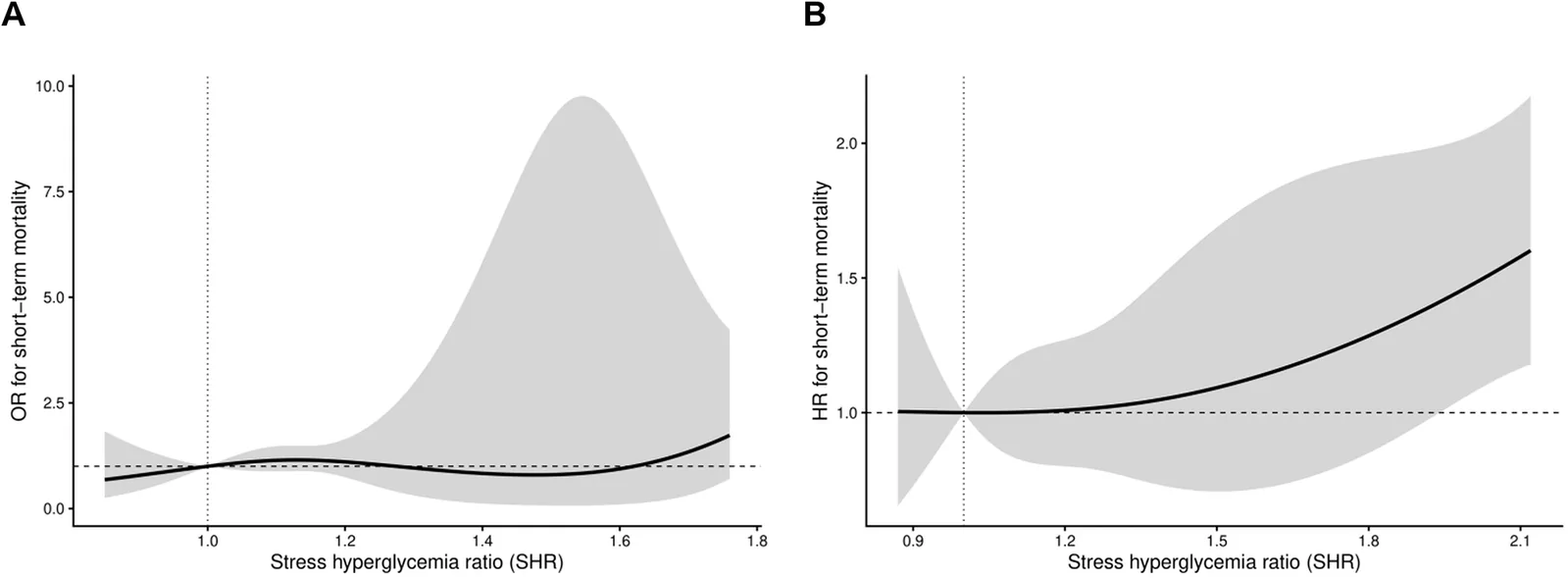
Restricted cubic spline dose-response curves for SHR and short-term mortality. **(A)** Odds ratio studies. **(B)** Hazard ratio studies.

The exploratory HR-RCS analysis included three studies (19, 21, 25). The overall association in the spline model was statistically significant (*P* = 0.013), but the nonlinearity test was not significant (*P* = 0.736). These results suggest an overall association between SHR and short-term mortality risk in HR studies, but the available data do not support a clear nonlinear dose-response relationship. Because the number of studies and dose points was limited, RCS results were used mainly to describe curve shape and should not be viewed as decisive evidence confirming or excluding nonlinearity. The HR dose-response curve is shown in Figure 3B. Nonlinear dose-response results are summarized in Table 4.

### 3.7 Exploratory analyses of low and high SHR

Because some original studies suggested that the association between SHR and mortality might not be purely monotonic, we conducted a separate exploratory analysis comparing low- and high-SHR categories with an intermediate SHR category. This analysis included only two studies18,23, because only these studies provided or allowed derivation of comparable low- and high-end contrasts relative to an intermediate SHR category. The pooled OR for the low-SHR category vs. the intermediate SHR category was 2.20 (95% CI: 0.08–57.69, *P* = 0.200, *I*^2^ = 4.8%). The pooled OR for the high-SHR category vs. the intermediate SHR category was 3.11 (95% CI: 0.64–15.15, *P* = 0.070, *I*^2^ = 0%). Both exploratory analyses pointed toward higher mortality risk at the low and high SHR ends, but confidence intervals were wide and neither estimate reached statistical significance. A potential U-shaped or J-shaped relationship therefore requires further validation. The comparisons from Qiao (18) were derived by transformation, with confidence intervals estimated approximately, and should be regarded only as exploratory evidence. Exploratory results are shown in Table 4, and the forest plots are shown in Supplementary Figure 1.

## 4 Discussion

Across the included studies, higher SHR was associated with greater short-term mortality, but the result depended on the effect measure and the amount of available information. The pooled HR was statistically significant and showed little detected heterogeneity; the pooled OR pointed upward but was imprecise, heterogeneous and not statistically significant. Linear dose-response analysis reached significance only for HRs. The spline models provided no clear evidence that a nonlinear model fitted better than a linear one. The two-study low/high analysis raised the possibility of excess risk at both extremes, but its wide confidence intervals limit it to hypothesis generation.

SHR addresses a specific limitation of admission glucose: the same acute glucose concentration can represent a different departure from baseline in two patients with different HbA1c levels. Roberts introduced SHR to scale acute glucose to the HbA1c-derived chronic glycemic background (13). In critical illness, relative hyperglycemia has been associated with in-hospital mortality (14), and a recent systematic review described SHR as a measure of acute glycemic deviation rather than absolute glycemia (9). That distinction is pertinent to respiratory critical illness, in which diabetes, infection, hypoxemia and organ dysfunction frequently coexist.

An SHR of 1 has a numerical meaning, but it should not be treated as a universal prognostic cut-off. By definition, SHR = 1 when acute-phase glucose equals the HbA1c-derived estimated average glucose (13, 15). Values above or below 1 indicate glucose above or below that estimated baseline; they do not, by themselves, define harmful or protective states. The original studies used different statistical references. Qiao used the lowest quartile (SHR ≤ 0.76); Bai used the lowest quartile and estimated an RCS minimum of 1.052; Ma used 1.000–1.299 as the reference category and located the spline minimum at 1.109; and Hu dichotomized SHR at an RCS-derived value of 1.215 (18–20, 23). Our dose-response models were likewise centered on each study's reference dose. Thus, an SHR of 1 is a useful interpretive anchor for relative glycemia, but it should not be regarded as a universally established normal value, prognostic threshold, or common reference category across the evidence base.

The label “severe respiratory disease” covers clinically and analytically distinct cohorts. The COPD evidence came from a diabetes-restricted ICU cohort in Chen and a broader critical-care COPD cohort in Qiao; their SHR groupings and mortality estimates were not directly equivalent (17, 18). Bai studied respiratory failure broadly, whereas Hu selected acute hypoxemic respiratory failure and used a data-derived threshold (19, 20). Infection-related cohorts were more varied still: Ma, Wang and Miao studied SCAP in different age groups and settings, while Mondal, Du and Aon examined COVID-19 using different designs, glucose measures and outcomes (16, 21–23, 25, 27). Liao's pulmonary embolism cohort represented a thromboembolic rather than primarily infectious or obstructive condition (24). The recurrence of higher mortality at higher SHR across several settings is notable, but it does not make the subtype-specific effect sizes interchangeable.

The OR-HR contrast is statistical as well as clinical. Logistic models estimate odds over a defined outcome window, whereas Cox models estimate the relative event hazard over follow-up. Pooling them would therefore combine different quantities. The OR summary had a large point estimate, a wide confidence interval and a prediction interval spanning the null. After Mondal was removed, heterogeneity fell sharply. That study differed from the other OR cohorts in its prospective single-center COVID-19 design, binary SHR threshold of 1.14, and inclusion of patients with diabetes, prediabetes or unknown glycemic status (22). These features are plausible contributors to the dispersion, although a leave-one-out analysis cannot identify the responsible factor. The HR studies were more alike in effect model and time-to-event interpretation, which helps explain their narrower pooled estimate; with only three studies, however, that precision should not be described as definitive.

The dose-response analyses should be read as a synthesis of grouped published data, not as a reconciliation of every curve reported by the source studies. The pooled HR slope rose with SHR, whereas the OR slope was not statistically significant. None of the pooled spline models improved on its linear counterpart. At the study level, Ma and Bai reported U-shaped curves with estimated minima of 1.109 and 1.052, respectively; Hu reported a sharper increase beyond 1.215; and Qiao reported nonlinearity across COPD quartiles (18–20, 23). Liao described a gradual linear increase in pulmonary embolism, while Du detected nonlinear components in critically ill patients with COVID-19 (21, 24). Differences in case mix, glucose sampling, SHR categories, reference groups and curve specification can generate different shapes. The pooled analysis therefore argues against claiming a common U-shaped, J-shaped or threshold relation; it does not exclude such relations within a particular population.

Low SHR still warrants attention, but its interpretation is less secure. A value below 1 means that acute-phase glucose is lower than the HbA1c-derived average; it does not reveal whether the reason is reduced intake, glucose-lowering treatment, impaired hepatic glucose output, limited nutritional reserve or organ dysfunction (9, 35). Ma observed higher in-hospital mortality below 1.000 than in the 1.000–1.299 reference group (23). The pooled low-end comparison was based on only two studies and had an extremely wide confidence interval. It therefore cannot establish a protective range near 1 or confirm excess risk at low SHR. Clinical interpretation must remain tied to diabetes status, treatment, nutrition, hepatic and renal function, and illness severity.

Several pathophysiological pathways may help explain the observed association. Severe respiratory diseases are often accompanied by hypoxia, infection, sympathetic activation and elevated glucocorticoid levels (1, 36). These factors can increase hepatic glucose output, insulin resistance and acute glucose fluctuations (37). Sustained or excessive stress hyperglycemia may then worsen oxidative stress, endothelial dysfunction, immune-cell impairment and coagulation abnormalities (9). In diabetic inpatients with pneumonia, Liu and colleagues found that higher SHR was associated with higher leukocyte count, neutrophil-to-lymphocyte ratio and C-reactive protein levels. Miao and colleagues also suggested possible reciprocal mediation between NLR and SHR (16, 26). In AECOPD, prior studies have linked HbA1c, the glycemic gap or HbA1c-adjusted glycemic variables to inflammation, acute respiratory failure, mechanical ventilation or mortality risk (38–42). These observations provide biological plausibility, but they do not establish that SHR lies on a causal pathway to death in respiratory critical illness. These findings also indicate that SHR should be interpreted within the broader clinical context, including age, concomitant organ dysfunction, cardiovascular comorbidity, coagulation status, and systemic inflammatory burden, rather than as an isolated prognostic marker.

Glucose and HbA1c obtained early after admission allow SHR to be calculated with routinely available measurements. The ratio offers individualized context for acute glycemia across patients with diabetes, prediabetes or unknown glycemic status. In Mondal's COVID-19 cohort, SHR discriminated mortality and adverse outcomes better than admission glucose; Aon also reported an association with a composite adverse outcome in hospitalized patients with diabetes and COVID-19 (22, 27). These observations do not justify a single SHR threshold or a uniform glucose-lowering strategy. SHR should be considered an adjunct for risk stratification, not a standalone therapeutic target.

The main strength of this study is its focused synthesis of SHR evidence in critically ill patients with respiratory diseases. Separating ORs from HRs reduced interpretive bias from pooling statistically different effect measures, and the combination of main-effect, linear dose-response, exploratory RCS and low/high SHR analyses provided a broader view of the exposure-outcome pattern.

This study also has several limitations. First, all included studies were observational. Although most original studies used multivariable adjustment, the covariates included in these models were not uniform across studies. In particular, the available study-level data did not permit harmonized adjustment for clinically relevant factors such as age, renal dysfunction or acute kidney injury, hepatic dysfunction or transaminase elevation, cardiovascular comorbidities such as heart failure, coagulation abnormalities including disseminated intravascular coagulation, and inflammatory or hematologic markers such as total white blood cell count, lymphocyte count, and interleukin-6, in addition to disease severity, glucocorticoid exposure, insulin treatment, nutritional status, and baseline glycemic state. Residual confounding from these factors therefore cannot be excluded. The findings should therefore be interpreted as associative evidence and do not establish a causal relationship between higher SHR and short-term mortality. Second, studies differed in disease type, diabetes prevalence, source and timing of acute-phase glucose measurement, timing of HbA1c measurement, SHR thresholds and reference-group definitions, and heterogeneity was high in OR analyses. These differences may affect effect estimates and between-study comparability. Third, some analyses included few studies. The RCS and low/high SHR exploratory analyses had limited statistical power and cannot fully exclude a true nonlinear relationship. Fourth, some studies came from MIMIC-IV or related databases. Although we tried to avoid double-counting overlapping populations in primary analyses, database studies may still differ in selection criteria and variable definitions. Fifth, individual patient data were unavailable, preventing harmonized covariate adjustment, unified SHR cutoffs, and further analyses of glycemic variability, insulin treatment, glucocorticoid therapy, nutritional status and changes in disease severity. Finally, fewer than 10 studies were included in each quantitative analysis, so publication bias could not be reliably assessed.

Future research should prioritize multicenter prospective cohort studies to define clinically useful SHR ranges across respiratory-disease subtypes, diabetes status and disease-severity strata. Studies using continuous glucose monitoring, glycemic variability, inflammatory markers and nutritional status may help clarify how acute metabolic stress relates to respiratory outcomes. For clinical use, SHR is best considered one component of a comprehensive risk-assessment system. Future studies should test whether SHR improves prediction beyond established severity scores and inflammatory markers. Future prospective studies should therefore collect individual-level data on these factors, including age, renal and hepatic function, cardiovascular comorbidity, coagulation status, and inflammatory or hematologic markers such as total white blood cell count, lymphocyte count and interleukin-6, to enable harmonized covariate adjustment and subgroup analyses.

## 5 Conclusion

This systematic review and meta-analysis suggest that higher SHR is associated with increased short-term mortality risk in critically ill patients with respiratory diseases, with stronger and more consistent evidence from HR-based analyses than from OR-based analyses. Current studies do not show that the RCS model outperforms the linear model, so the evidence is insufficient to confirm a clear nonlinear pattern. Exploratory analyses based on a small number of studies suggest that both low and high SHR may be associated with increased risk, but the estimates are imprecise. SHR may provide Supplementary material for early risk stratification after admission, but its optimal threshold, incremental predictive value and applicability across respiratory-disease subtypes require prospective validation.

## Data Availability

The data analyzed in this study are subject to the following licenses/restrictions: No legal or ethical restrictions apply to the dataset. However, the extracted study-level dataset has not been deposited in a public repository and is available from the first author upon reasonable request. Requests to access the dataset should be directed to Wenjie Zhang at jerryzwj0628@163.com.
