# Supplementary material for: Stress hyperglycemia ratio and short-term mortality in critically ill patients with respiratory diseases: a systematic review and dose-response meta-analysis
Source: Front Med (Lausanne). 2026 Oct 8;13:1937012. doi: 10.3389/fmed.2026.1937012 (PMC13687381; doi:10.3389/fmed.2026.1937012)
Supplement: Supplementary file 1 [file Table1.docx]

**Supplementary Table S1. GRADE assessment of certainty of evidence for the association between stress hyperglycemia ratio and short-term mortality in critically ill patients with respiratory diseases**

| Evidence body | No. of studies | Effect estimate | Risk of bias | Inconsistency | Indirectness | Imprecision | Publication bias | Certainty of evidence |
| --- | --- | --- | --- | --- | --- | --- | --- | --- |
| High/elevated SHR vs reference (OR) | 4 | OR 2.72 (95% CI 0.74–9.93) | Seriousᵃ | Seriousᵇ | Not serious | Seriousᶜ | Undetectedᵈ | Very low ⊕◯◯◯ |
| High/elevated SHR vs reference (HR) | 3 | HR 1.87 (95% CI 1.71–2.05) | Seriousᵃ | Not serious | Not serious | Not serious | Undetectedᵈ | Moderate ⊕⊕⊕◯ |
| SHR per 0.1-unit increase (OR) | 3 | OR 1.03 (95% CI 0.97–1.10) | Seriousᵃ | Not serious | Not serious | Seriousᶜ | Undetectedᵈ | Low ⊕⊕◯◯ |
| SHR per 0.1-unit increase (HR) | 3 | HR 1.05 (95% CI 1.02–1.08) | Seriousᵃ | Not serious | Not serious | Not serious | Undetectedᵈ | Moderate ⊕⊕⊕◯ |

**Abbreviations:** SHR, stress hyperglycemia ratio; OR, odds ratio; HR, hazard ratio; CI, confidence interval.

**GRADE certainty levels:** High ⊕⊕⊕⊕; Moderate ⊕⊕⊕◯; Low ⊕⊕◯◯; Very low ⊕◯◯◯.

**a. Risk of bias:** Downgraded one level because, although multivariable-adjusted estimates were generally used, adjustment variables were not harmonized across studies and residual confounding from disease severity, organ dysfunction, diabetes status, treatment exposure, and other prognostic factors could not be excluded.

**b. Inconsistency:** Downgraded one level because substantial heterogeneity was observed in the OR analysis (I²=86.4%). However, effects were directionally consistent, and heterogeneity decreased substantially after exclusion of the Mondal study.

**c. Imprecision:** Downgraded one level because the confidence interval was wide and included both little/no association and potentially meaningful increases in mortality risk.

**d. Publication bias:** Publication bias was considered undetected because fewer than 10 studies contributed to each quantitative synthesis; formal funnel plot or regression-based assessments were therefore not reliable.
